# Prevalence and viral loads of polyomaviruses BKPyV, JCPyV, MCPyV, TSPyV and NJPyV and hepatitis viruses HBV, HCV and HEV in HIV-infected patients in China

**DOI:** 10.1038/s41598-020-74244-0

**Published:** 2020-10-13

**Authors:** Xianfeng Zhou, Kenji Nakashima, Masahiko Ito, Xiaoling Zhang, Satoshi Sakai, Changhua Feng, Huabao Sun, Haiying Chen, Tian-Cheng Li, Tetsuro Suzuki

**Affiliations:** 1 The Collaboration Unit for Field Epidemiology of State Key Laboratory for Infectious Disease Prevention and Control, Jiangxi Provincial Key Laboratory of Animal-Origin and Vector-Borne Diseases, Nanchang Center for Disease Control and Prevention, Nanchang, 330038 China; 2 Department of Virology and Parasitology, Hamamatsu University School of Medicine, Hamamatsu, 431-3192 Japan; 3 Department of Molecular Biology, Hamamatsu University School of Medicine, Hamamatsu, 431-3192 Japan; 4 Department of Clinical Laboratory, Affiliated Infectious Diseases Hospital of Nanchang University, Nanchang, 330002 China; 5 Department of Virology II, National Institute of Infectious Diseases, Musashi-Murayama, Tokyo, 208-0011 Japan

**Keywords:** Infectious diseases, Hepatitis, HIV infections, Viral infection

## Abstract

Human polyomaviruses (PyVs) and hepatitis viruses are often more prevalent or persistent in human immunodeficiency virus (HIV)-infected persons and the associated diseases are more abundant than in immunocompetent individuals. Here, we evaluated seroreactivities and viral loads of human PyVs and hepatitis viruses in HIV/AIDS patients and the general population in China in the combination antiretroviral therapy (cART) era. A total of 810 HIV-1-infected patients and age- and sex-matched HIV-negative individuals were enrolled to assess seroprevalence of PyVs BKPyV, JCPyV, MCPyV, TSPyV, and NJPyV and hepatitis viruses HBV, HCV, and HEV. 583 (72%) patients received cART, and among them, 31.2% had undetectable HIV RNA. While no significant difference was observed in prevalence of anti-PyV antibodies between HIV-positive and -negative groups, serum DNA positivity and DNA copy level of MCPyV were higher in the HIV-positive group. Among HIV-infected patients, BKPyV DNA positivity was significantly higher in patients with CD4 + cell counts < 200 cells/mm^3^ compared to those with CD4 + cell counts > 500 cells/mm^3^, suggesting possible reactivation caused by HIV-induced immune suppression. Higher HBV and HCV seropositivities but not HEV seropositivity were also observed in the HIV-positive group. Further correlation analyses demonstrated that HBV and HEV are potential risk factors for increased prevalence of PyV infection.

## Introduction

Numerous viruses including human polyomaviruses (PyVs) and hepatitis viruses are more prevalent or persistent in human immunodeficiency virus (HIV)-infected individuals, and the associated diseases are more abundant in this population than in immunocompetent individuals^1–3^. PyVs are small, non-enveloped double-stranded DNA viruses. To date, 14 human PyVs have been identified since the discovery of the first two human PyVs, BK virus (BKPyV) and JC virus (JCPyV)^4–6^. Patients with BKPyV and JCPyV infection are usually asymptomatic, but immunosuppression can lead to reactivation, which is associated with serious diseases such as BKPyV-induced nephropathy or JCPyV-induced leukoencephalopathy. The association of Merkel cell polyomavirus (MCPyV) with Merkel cell carcinoma in immunocompromised individuals has also been revealed^7^. Trichodysplasia spinulosa-associated polyomavirus (TSPyV) was discovered in skin lesions of immunosuppressed patients with the rare disease trichodysplasia spinulosa^8^.

While it is considered that PyV DNA levels correlate with clinical outcomes and viral reactivation in AIDS patients, findings from molecular epidemiological studies remain controversial. For instance, viral loads of human PyVs including MCPyV do not significantly differ between HIV-positive and -negative men^9^. Regarding New Jersey polyomavirus (NJPyV), which was discovered in 2014 in vascular endothelial cells of a pancreatic transplant recipient, only a few serological surveys on this virus have been reported in healthy populations and no study on HIV-positive individuals has been described to date^4,10,11^.

Viral hepatitis has emerged as an important public health issue globally and is characterized by high prevalence and high burden of morbidity and mortality.

Because HIV, hepatitis B virus (HBV), and hepatitis C virus (HCV) share transmission routes, a considerable number of HIV-positive patients (5–20%) were found to be co-infected with HBV or HCV^12,13^. In general, this co-infection is associated with higher levels of HBV DNA or HCV RNA, accelerated progression of liver disease, in particular hepatic fibrosis, and increased liver-associated mortality compared with HBV or HCV mono-infection. Global distribution of hepatitis E virus (HEV), a prevalent fecal–oral transmitted agent, exhibits distinct epidemiological patterns that are dependent on socioeconomic conditions, sanitation level, blood transfusion, and occurrence of zoonotic transmission^14^. Although a high proportion of hepatitis E cases have self-limiting asymptomatic or subclinical infections, immunosuppression such as in HIV-infected patients may modify the pathogenesis and clinical impact of this emerging disease, including development of chronic infection.

The global expansion in the use of combination antiretroviral therapy (cART) has reduced HIV-related mortalities and HIV incidence. While many studies to date have investigated the epidemiology of human PyVs or hepatitis viruses in HIV-infected patients separately, little has been reported on the prevalence of both PyVs and hepatitis viruses in the same HIV-positive cohort in the cART era. In this study, we evaluated seroreactivities and viral loads of the PyVs and hepatitis viruses indicated above in HIV/AIDS patients and the general population in China. Our findings may help in analyses of the possible correlation between prevalence of PyVs and hepatitis viruses in these cohorts.

## Results

### Characteristics of study participants

Among 810 HIV-positive patients, 72% had received cART at the time of sampling and survey (Table 1). Serum HIV RNA load significantly decreased after cART treatment for longer than 6 months (Supplementary Fig. 1A).

**Table 1:** Demographic information of the study cohorts.

	HIV-positive patients n (% or IQR)	Control persons n (% or IQR)	*P* value
**Total**	810 (100)	810 (100)	
Male versus female	6.1:1	7.3: 1	0.18
Median age, years	41 (26–54)	38 (25–49)	
**Age, years**			0.23
18–20	45 (5.5)	59 (7.3)	
21–30	256 (31.6)	260 (32.1)	
31–40	143 (17.7)	149 (18.4)	
41–50	111 (13.7)	175 (21.6)	
51–60	122 (15.1)	76 (9.4)	
61–85	133 (16.4)	91 (11.2)	
**Ethnicity**			
Han	775 (95.7)	793 (97.9)	0.11
Yi	22 (2.7)	9 (1.1)	
Uygur	8 (1.0)	3 (0.6)	
others	5 (0.6)	5 (0.4)	
**cART**			
Yes versus no	2.6:1	N/A	N/A

*IQR* interquartile range, *N/A* not applicable; *P* vaule was caculated using X^2^ test.

As shown in Supplementary Fig. 2, 104 (12.8%) and 67 (8.3%) of patients in the HIV-positive group, respectively, were co-infected with HBV and HCV. Ten (1.2%) of the HIV/AIDS patients had HIV–HBV–HCV triple infection, and 136 (16.8%) of the patients were co-infected with one or more PyVs. Furthermore, a positive correlation was observed between HIV and HBV levels in sera of co-infected patients receiving cART (*r* = 0.32, *P* = 0.008) but not in cART-naïve patients (*r* = 0.19, *P* = 0.412), indicating that cART is potentially effective for HBV suppression. No correlation was observed between HCV and HIV levels in co-infected patients, regardless of cART use (Supplementary Fig. 1B).

Rates of three HIV infection routes, heterosexual transmission (64.4%), men who have sex with men (MSM; 28.7%), and intravenous drug use (IDU; 6.9%), were collected by questionnaire survey. The frequency of IDU was higher in both HCV-positive groups (with and without HBV co-infection) compared to the HCV-negative group as well as all HIV-positive patients, indicating high risk of HCV transmission by potential contamination during drug injection (Supplementary Fig. 2).

### Prevalence of anti-PyV IgG antibodies and PyV DNA in HIV-positive and -negative populations

**Figure 1:**
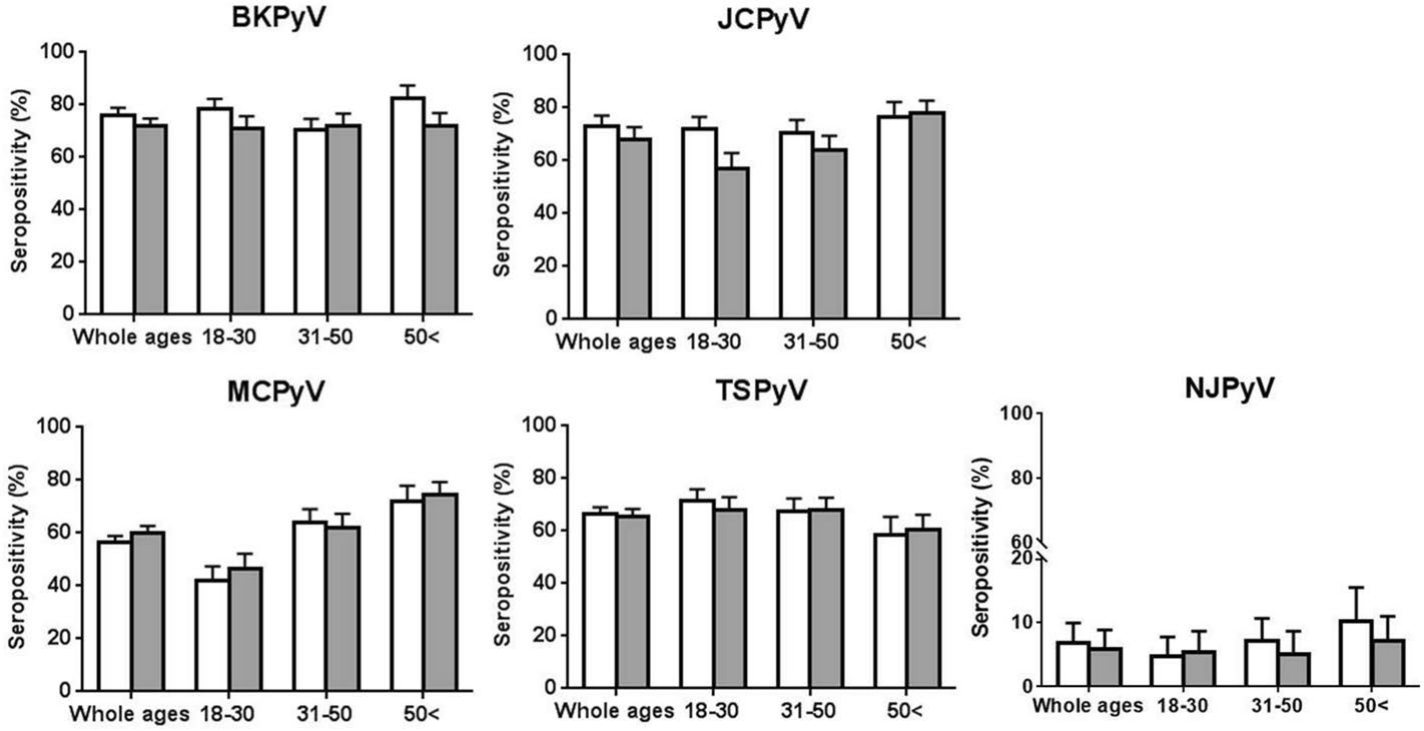
Seroprevalence of anti-BKPyV, anti-JCPyV, anti-MCPyV, anti-TSPyV and anti-NJPyV IgG antibodies among HIV-positive (gray bar) and HIV-negative (white bar) populations. Error bars represent 95% confidence intervals.

Prevalence of anti-PyV IgG antibodies for BKPyV, JCPyV, MCPyV, NJPyV, and TSPyV was measured by individual VLP-based ELISAs^4^. There was no significant difference in the overall seroprevalence of the five PyVs between HIV-positive and HIV-negative groups: 71.6% versus 76.2% for BKPyV, 65.8% versus 71.7% for JCPyV, 60.1% versus 56.5% for MCPyV, 65.4% versus 65.8% for TSPyV, and 5.8% versus 6.8% for NJPyV, respectively (Fig. 1). Furthermore, seropositivity rates for men and women were not significantly different for each PyV (data not shown). Pair correlation analyses showed no correlation between seroreactivities against individual PyVs regardless of HIV status, indicating high specificity of VLP-based ELISAs used in this study (Supplementary Table 2). Study participants were also categorized into three age groups: 18–30 years, 31–50 years, and > 50 years (Fig. 1). While seropositivities of anti-BKPyV and JCPyV antibodies were high in all age groups, the positivity of anti-MCPyV antibody increased according to age and the highest prevalence was observed in the > 50 age group. In contrast, the highest prevalence of anti-TSPyV antibody was detected in the 18–30 age group. Samples with high OD values (> 1 of ELISA data for anti-TSPyV antibody) were more frequently observed in the 18–30 age group compared to the 31–50 and > 50 age groups (Supplementary Fig. 3). Overall OD values for anti-NJPyV antibody were quite low among all study participants in contrast to those for the other four PyVs (Supplementary Fig. 3).

**Table 2:** Viral DNA/RNA positivity between CD4 + cell count < 200 and CD4 + cell count > 500 in HIV-infected patients.

	CD4 < 200 (N = 211)	CD4 > 500 (N = 180)	*P* value
**Positivity, n (%)**			
BKPyV DNA	23 (10.9)	9 (5.0)	0.002
JCPyV DNA	8 (3.8)	6 (3.3)	0.808
MCPyV DNA	15 (7.1)	11 (6.1)	0.693
TSPyV DNA	3 (1.4)	2 (1.1)	0.785
HBV DNA	30 (14.2)	14 (7.8)	0.045
HCV RNA	4 (1.9)	8 (4.4)	0.145

*P* value was calculated using Pearson’s chi square test.

**Figure 2:**
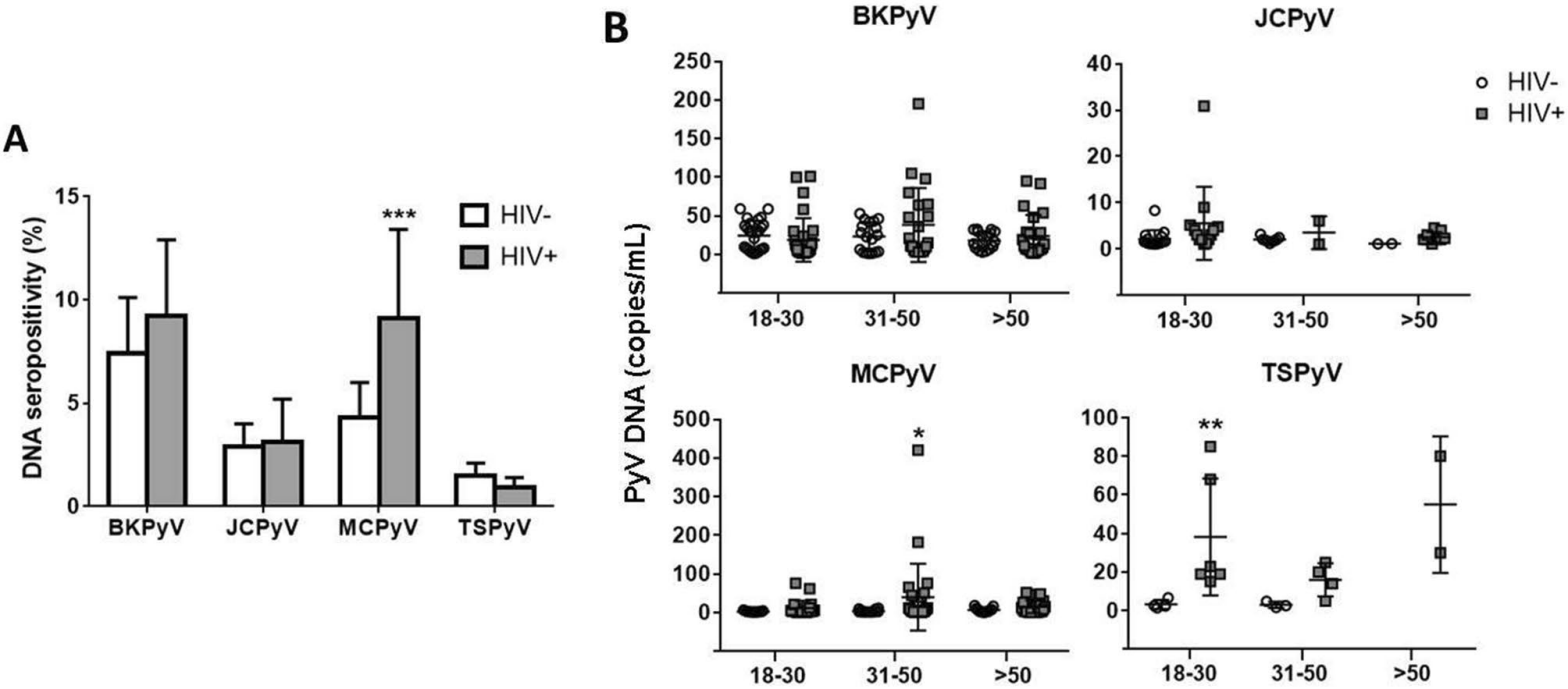
Positivities (**A**) and copies (**B**) of viral DNAs for BKPyV, JCPyV, MCPyV and TSPyV in three age groups of HIV-positive and HIV-negative populations. Pearson’s chi square test was used to compare DNA positivities in (**A**) and Mann–Whitney U test was used for group comparison in (**B**). Error bars in (**A**) and (**B**), respectively, represent 95% confidence intervals and mean with SD (*P < 0.05; **P < 0.01; ***P < 0.001).

Next, serum PyV DNA positivities were compared between HIV-positive and -negative groups (Fig. 2A). MCPyV DNA positivity in the HIV-positive group (9.1%) was significantly higher than that in the HIV-negative group (4.2%) (*P* < 0.001). In the 31–50 age group, serum MCPyV DNA load was significantly higher in HIV-positive participants compared to that in HIV-negative controls (40 ± 87 versus 4 ± 4 copies/mL, *P* < 0.05) (Fig. 2B). While the TSPyV DNA positivity rate in the overall cohort was very low (0.9–1.5%) (Fig. 2A), TSPyV viral load overall was higher with HIV infection (*P* < 0.05). In the 18–30 age group, TSPyV DNA loads in HIV-positive participants were significantly higher than those in HIV-negative controls (*P* < 0.01) (Fig. 2B).

### Prevalence of hepatitis viruses in HIV-positive and -negative populations

**Figure 3:**
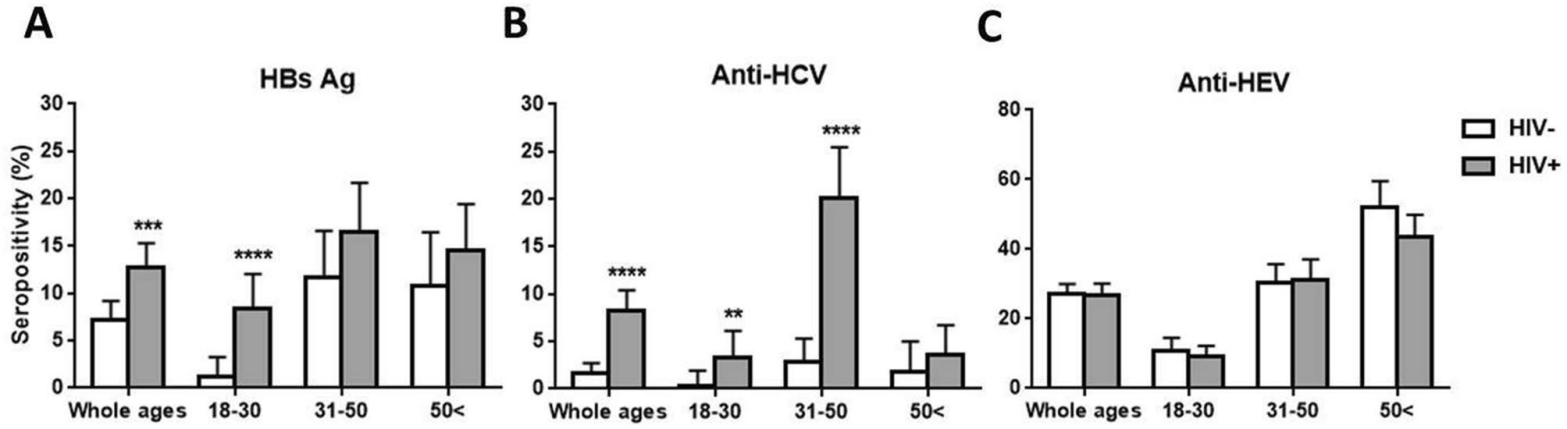
Seroprevalence of HBs Ag, anti-HCV and anti-HEV IgG in HIV-postivie and HIV-negative populations. Pearson’s chi square test was used to compare seropositivities. Error bars represent 95% confidence intervals (**P < 0.01; ***P < 0.001; ****P < 0.0001).

In the HIV-negative group, prevalence rates of serum HBs antigen, anti-HCV antibody, and anti-HEV antibody were 7.2%, 1.6%, and 27.3%, respectively (Fig. 3). Prevalence of HBV and HCV infection in the HIV-positive population was higher than that in the HIV-negative population. In particular, the HBs antigen positivity rate in the 18–30 age group of the HIV-positive population was approximately sixfold higher than that in the corresponding control group (8.3% versus 1.3%, *P* < 0.0001) (Fig. 3A). Anti-HCV seropositivity was markedly higher in the 18–30 and 31–50 age groups, respectively, compared with the control groups (3.3% vs. 0.3%, *P* < 0.01, and 20.1% vs. 2.8%, *P* < 0.0001) (Fig. 3B). Co-infection with HBV and HCV was observed in the HIV-positive group (Supplementary Fig. 2) but not in the control group (data not shown). The liver is one of the potential targets of HIV infection and various liver diseases are observed in HIV-infected patients. As expected, both alanine transaminase (ALT) and aspartate transaminase (AST) levels in patients with HIV-HBV-HCV triple infection were higher than those in HIV-positive patients co-infected either with HBV or HCV (Supplementary Fig. 4). Furthermore, the prevalence of liver fibrosis in co-infection with HBV or HCV was significantly higher; for example, FIB-4 > 3.25 was 9.9% for HIV mono-infection, 23.5% for HIV-HBV co-infection, and 16.1% for HIV–HCV co-infection (Supplementary Table 3). Seroprevalence of anti-HEV antibody was not significantly different between HIV-positive and -negative groups (Fig. 3C). These results suggest that HIV infection positively correlates with HBV and HCV infection but not with HEV infection.

### Correlation between CD4 + cell counts and DNA positivities of co-infected viruses in the HIV-positive group

CD4 + cell count is used as a prognostic marker of HIV/AIDS disease progression. According to the previous global guideline, the initiation of cART is based on CD4 + count < 350 cells/mm^3^. Since 2016, WHO has recommended that cART be initiated in everyone living with HIV, regardless of CD4 + cell count. In the present study, 26.0% and 22.2% of HIV-positive participants had CD4 + counts < 200 cells/mm^3^ and > 500 cells/mm^3^, respectively (Table 2). Among PyVs and hepatitis viruses tested, BKPyV and HBV DNA positivities among HIV/AIDS patients with CD4 + counts < 200 cells/mm^3^ were significantly higher than those with CD4 + counts > 500 cells/mm^3^ (*P* = 0.045 for HBV; *P* = 0.002 for BKPyV).

### Possible correlation between prevalence of PyVs and hepatitis viruses

**Figure 4:**
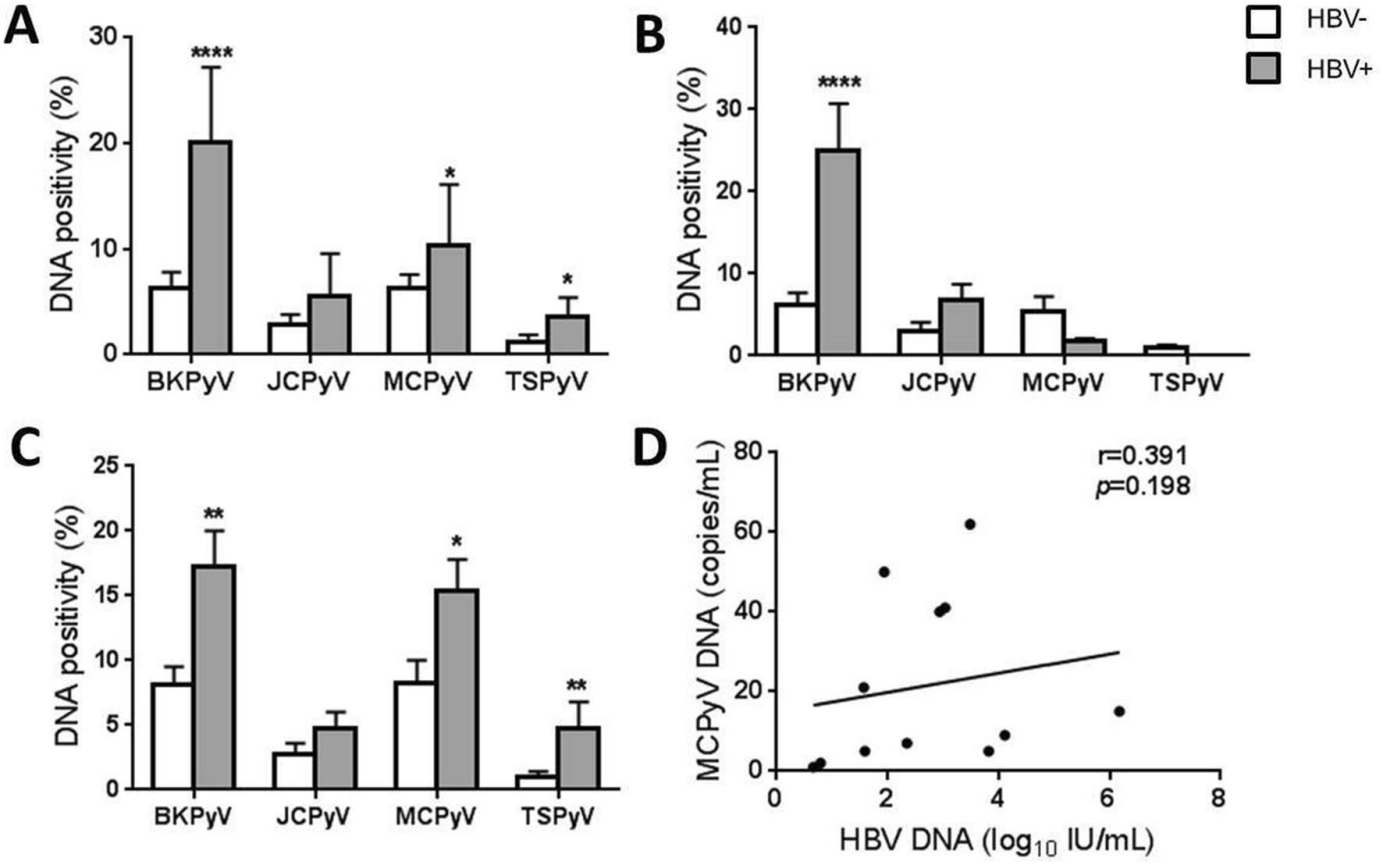
DNA positivities of BKPyV, JCPyV, MCPyV and TSPyV in HBsAg-negative (White bar) and HBV-positive (Gray bar) individuals. (**A**) Entire study cohort; (**B**) HIV-negative population; (**C**) HIV-positive population. Pearson’s chi square test was used to compare DNA positivities. Error bars represent 95% confidence intervals (*P < 0.05; **P < 0.01; ****P < 0.0001). (**D**) Correlation of serum HBV and MCPyV viral levels. Pearson correlation test was used for correlation analysis.

We next evaluated whether prevalence of BKPyV, JCPyV, MCPyV, and TSPyV is associated with hepatitis virus infection. Among the study population, 104 (12.8%) and 60 (7.4%) HBV-positive participants were included in the HIV-positive and -negative groups, respectively. Serum DNA positivities of all PyVs tested were higher in the HBV-positive group compared to those in the HBV-negative group. In particular, HBV infection significantly correlated with DNA positivities of BKPyV (*P* < 0.0001), MCPyV (*P* < 0.05), and TSPyV (*P* < 0.05) (Fig. 4A). Similar positive associations between HBV infection and BKPyV, MCPyV, or TSPyV were observed in the HIV-positive group (Fig. 4C). Higher DNA positivities of BKPyV and JCPyV among HBV-positive patients were also seen in the HIV-negative group (Fig. 4B). In addition, serum HBV DNA level positively correlated with MCPyV DNA level (*r* = 0.391, *P* < 0.01) (Fig. 4D) but not with BKPyV DNA level (data not shown). In contrast, HCV infection was not markedly associated with PyV DNA positivities (Supplementary Fig. 5).

We further analyzed the possible correlation of prevalence of anti-PyV IgG antibodies for BKPyV, JCPyV, MCPyV, TSPyV, and NJPyV with that of anti-HEV antibody (Table 3). Approximately 27% of participants (218 participants in the HIV-positive group and 221 participants in the HIV-negative group) were positive for anti-HEV IgG. MCPyV seropositivity was significantly higher in the anti-HEV-positive population compared with the anti-HEV-negative population, regardless of HIV status. In HIV-positive individuals, JCPyV seropositivity was also significantly associated with HEV seropositivity (*P* = 0.049). Collectively, it is likely that PyV infection is associated with both HBV and HEV infection, suggesting multiple risk factors involved in PyV transmission.

**Table 3:** Association between Anti-PyV IgG- and Anti-HEV IgG Seroprevalence.

Anti-PyV IgG	HIV ( +)			HIV (−)		
	Anti-HEV IgG + (N = 218) n (%)	Anti-HEV IgG- (N = 592) n (%)	*P* value	Anti-HEV IgG + (N = 221) n (%)	Anti-HEV IgG − (N = 589) n (%)	*P* value
BKPyV	170 (78.0)	451 (76.2)	0.591	178 (80.5)	476 (80.8)	0.930
JCPyV	156 (71.6)	380 (64.2)	0.049	152 (68.7)	441 (74.9)	0.081
MCPyV	155 (71.1)	358 (60.4)	0.005	142 (64.2)	317 (55.8)	0.030
TSPyV	142 (65.1)	391 (66.1)	0.809	142 (65.5)	404 (69.6)	0.241
NJPyV	18 (8.3)	29 (4.9)	0.070	14 (6.3)	41 (7.0)	0.752

*P* value was calculated using Pearson’s chi square test.

## Discussion

Seroepidemiological investigation of human PyVs not only in the general population but also in immunocompromised individuals has been extensively reported worldwide. However, to our knowledge, this is the first study to demonstrate the prevalence of serum IgG antibodies against various PyVs and their DNAs in the HIV-infected population, in addition to comparative analysis with the general population cohort in the same geographic region. In this study, serum DNA positivity and DNA copy level of MCPyV in the HIV-positive group were higher than those in the HIV-negative group (Fig. 2). In the HIV-positive group, BKPyV DNA positivity was significantly higher in patients with CD4 + counts < 200 cells/mm^3^ compared to those with CD4 + counts > 500 cells/mm^3^ (Table 2). It is thus likely that immunosuppression observed in HIV/AIDS patients possibly facilitates PyV reactivation. It has been reported that the overall incidence of MCPyV-related carcinoma in HIV/AIDS patients was 13-fold higher than that in the general population^15^. TSPyV has been discovered in trichodysplasia spinulosa, a rare skin disease that occurs exclusively in immunocompromised patients, such as individuals undergoing organ transplantation. Nevertheless, TSPyV prevalence in HIV/AIDS patients has been poorly characterized to date. Here, our study demonstrated that, although TSPyV DNA positivity rates in the study populations were low (0.9–1.5%) compared to BKPyV, JCPyV, and MCPyV rates, TSPyV DNA levels in each age group of HIV-positive participants seem to be higher than the respective counterparts of HIV-negative participants (Fig. 2B).

In contrast to serum viral DNA analyses, no significant difference was observed in the overall prevalence of anti-PyV antibodies to the five PyVs tested between HIV-positive and -negative groups (Fig. 1), While seroreactivities might be impaired in the subset of HIV + individuals with low CD4 count and therefore the interpretation of the results should be limited, it appears that anti-PyV IgGs are potentially induced and maintained by antigenic stimulation through primary and latent infection with PyVs regardless of HIV infection. Age-dependent trends in IgG seroprevalence vary by PyVs (Fig. 1, Supplementary Fig. 3). For BKPyV and JCPyV, seropositivities and antibody levels were comparable among each age group. Conversely, anti-MCPyV antibody positivity increased with age and the highest prevalence was observed in the > 50 age group. Furthermore, the highest prevalence of anti-TSPyV antibody was detected in the 18–30 age group (Fig. 1) and its antibody levels (OD values) appeared to decrease with increasing age (Supplementary Fig. 3)^16–19^. Despite these 4 kinds of PyVs are considered to be present ubiquitously in the general population, it is likely that patterns of exposure to each virus are not consistent and some viruses such as TSPyV are possibly cleared with increasing ages, leading to difference in their incidence and persistence rates dependent on the PyVs. In contrast to BKPyV, JCPyV, MCPyV, and TSPyV, NJPyV seroprevalence was very low in our population. Similar findings on NJPyV seroprevalence were reported in the general population from the Netherlands (5.2%)^20^ and Japan (1.2%)^4^, suggesting low circulation of NJPyV in human societies.

We also evaluated the seroprevalence of HBV, HCV, and HEV as well as DNA/RNA positivity of HBV and HCV in the same study cohorts. HBV and HCV are bloodborne viruses and share common transmission routes with HIV. As expected, higher HBV and HCV seropositivities, but not HEV seropositivity, were observed in the HIV-positive group compared to the HIV-negative group (Fig. 3). Previous studies with matched controls reported that HIV infection was not identified as a risk factor for HEV infection^21–23^. Co-infection of HBV or HCV with HIV has been associated with increased risk of progression to liver disease and reduced survival. In HIV/AIDS patients, both ALT and AST levels were significantly higher with HBV and/or HCV co-infection (Supplementary Fig. 4) and the rate of liver fibrosis was significantly enhanced in patients with HBV or HCV (Supplementary Table 3). Ding et al. found that old age and male sex, as well as HBV or HCV co-infection, were positive predictors of fibrosis progression for HIV-infected patients^24^. HBV and HCV not only cause liver diseases but also result in failure of immunological recovery in HIV-positive patients^3,13^. In our study cohort, positivity of HBV DNA but not of HCV RNA among HIV-positive patients with CD4 + counts < 200 cells/mm^3^ was significantly higher than those with CD4 + counts > 500 cells/mm^3^ (Table 2). Findings of a positive correlation between HIV load and HBV, but not HCV, in patients treated with cART (Supplementary Fig. 1B) indicate that the ability to control HBV infection by introduction of nucleoside/nucleotide analogs with activity against HBV has significantly increased in HIV co-infected patients.

The prevalence of HIV co-infection either with HBV or HCV differs by geographic regions in China, with prevalence rates of 8.7–14.4% and 5.7–41.8% for HBV–HIV and HCV–HIV co-infection, respectively^25^. In this study, 12.8% and 8.3% of HIV/AIDS patients had HBV and HCV infections, respectively, including 1.2% of patients who had HBV–HCV–HIV triple infection.

While this study was primarily designed to elucidate the prevalence of human PyVs and hepatitis viruses, the data obtained enabled us to explore the possible correlation between the prevalence of PyVs and hepatitis viruses in our cohorts. We found that DNA positivities of all PyVs tested were higher in the HBV-positive group than those in the HBV-negative group among HIV-positive participants. Furthermore, serum HBV DNA level sera positively correlated with MCPyV DNA level (Fig. 4). While immune suppression induced by HIV infection potentially has an impact on propagation of MCPyV and HBV, any direct interaction between the two viruses during their infection/replication cycles has not been reported to date. The prevalence of anti-MCPyV IgG was significantly higher in the anti-HEV-positive group compared with the anti-HEV-negative, regardless of HIV infection status. In HIV-positive individuals, positivity for anti-JCPyV IgG was also significantly associated with HEV seropositivity (Table 3). Not only fecal–oral, oral, and respiratory routes but other routes such as bloodborne routes are implicated in the transmission of human PyVs^26,27^. Further association analysis indicated that HBsAg positivity was significantly associated with seroprevalence of BKPyV, JCPyV, MCPyV and TSPyV (*P* < 0.05). A higher prevalence of anti-MCPyV IgG was also observed in anti-HEV positive group (*P* < 0.05) (Supplementary Table 4). Although HEV vaccine (Hecolin) was available since late 2012 in China, it appears that the public are currently unware of the importance of the HEV vaccine and are poorly accepting this vaccine. In fact the number of hepatitis E cases reported after the vaccine licensing had remained at similar high level as found before the licensing^28^. It is thus likely that the extension of vaccine influence on HEV seroprevalence in this study was limited. Collectively, our findings demonstrated the possible relation between HBV and HEV with PyVs, suggesting that HBV and HEV are potentially risk factors for an increase in seroprevalence and incidence of PyV infection. Further studies to address possible biological interaction between hepatitis viruses and PyVs are required.

In conclusion, we provide an update on the prevalence and viral load of both human PyVs and hepatitis viruses in HIV-infected patients in China in the cART era. Viral levels of some human PyVs such as BKPyV and MCPyV in the HIV-positive population were potentially higher, which may facilitate reactivation of PyVs caused by immunosuppression. We confirmed that co-infection of HBV or HCV with HIV was associated with an increased risk of progression of liver disease. It is also likely that infection with HBV or HEV affects the natural history of PyV infection. Given the cytolytic and oncogenic potential of PyVs, it is important to further elucidate factors associated with prevalence and persistence of human PyV infection.

## Subjects and methods

### Study population

A total of 810 HIV-1-infected individuals (695 males and 115 females) and the same numbers of age- and gender-matched healthy (HIV-negative) controls (713 males and 97 females) were enrolled. The HIV-positive individuals were selected from five designated hospitals in Jiangxi, China from 2015 to 2016. Subsequently, the age- and gender-matched controls were randomly selected from nine resident communities in the same province. The median ages of HIV-positive and control groups, respectively, were 41 years (range 18–85 years) and 38 years (range 18–80 years). HIV-infected participants were positive for anti-HIV antibody and HIV-1 antigen. After the participants had provided informed consent, serum samples were collected and frozen prior to analyses. Demographic data were also collected (Table 1). Over 95% of participants were Han Chinese, which is a predominant ethnic population in China. This study was approved by the Institutional Review Board of the Nanchang Center for Disease Control and Prevention (Approval No. 20160301) and carried out in accordance with Declaration of Helsinki.

### Detection of anti-PyV antibodies by VLP-based ELISA

ELISAs to detect anti-IgG antibodies against virus-like particles (VLPs) of BKPyV, JCPyV, MCPyV, TSPyV and NJPyV were developed as described^4^. Briefly, 96-well microplates were respectively coated with the purified VLPs and incubated overnight at 4 °C. The wells were blocked with 5% skim milk dissolved in 10 mM phosphate-buffered saline containing 0.05% Tween 20 (PBS-T). After washing four times with PBS-T, diluted serum samples were added and incubated for 1 h. The wells were washed as indicated and then incubated with horseradish peroxidase-conjugated goat anti-human IgG (H + L) for 1 h. The plates were incubated for 1 h and washed four times. The substrate orthophenylenediamine and H_2_O_2_ were added, followed by adding 50 μl of 4 N H_2_SO_4_ to each well. Absorbance was measured at 492 nm. Histograms of optical density (OD) values indicating the levels of anti-PyV IgGs obtained from ELISA revealed bimodal distributions of seroreactivity against each PyV. The cut-off points for their seropositivity were thus defined as the mean of the lower distribution plus threefold standard deviation and were determined to be 0.25, 0.23, 0.19, 0.17 and 0.23 for BKPyV, JCPyV, MCPyV, TSPyV and NJPyV, respectively.

### Detection of anti-HIV, HCV and HEV antibodies and HBs antigen

Commercial ELISA kits (Beijing Wantai Biological) were used to detect HBs antigen, anti-HCV antibody, anti-HEV IgG antibody and anti-HIV IgG antibody in sera. Samples positive for anti-HIV antibody were subjected to confirmatory HIV testing using an HIV Blot 2.2 WB kit (MP Biomedicals). Positive results in the western blotting test were defined in terms of the detection of Env (gp160/gp41 and gp120) and Gag (p17, p24, p55), or Env (gp160/gp41 and gp120) and Pol (p31, p51, p66) proteins.

### Quantification of viral nucleic acids

To determine viral DNAs and RNAs, total nucleic acids were extracted from 200 μl sera using TIANamp Virus DNA/RNA Kit (TIANGEN Biotech). For quantitative real-time PCR, DNA of BKPyV, JCPyV, MCPyV and TSPyV were amplified using specific primers (Supplementary Table 1) in the presence of Thunderbird SYBR qPCR Mix (TOYOBO) on ABI 7300 (Applied Biosystems) according to manufacturer’s instruction. After 1 min denaturation at 95 °C, samples were subjected to 40 cycles at 95 °C for 15 s and 60 °C for 1 min. Data obtained were normalized to the amount of whole viral genome of each PyV, respectively, derived from pBR322-BKPyV (LC029411), JCPyV-mad1-case6-RR (LC164353), pTA22-MCPyV (FJ464337) and pCR2.1-TSPyV (AB873001). The PyV DNA copy numbers were calculated based on the standard curve that was generated by applying the tenfold diluted series of the purified plasmids. HBV DNA, HCV RNA and HIV RNA were quantitatively determined using HBV PCR Kit (DAAN gene), HCV PCR Kit (DAAN gene), and COBAS AmpliPrep/COBAS TaqMan HIV-1 Test, v2.0 (Roche Diagnostics), respectively.

### CD4 + T-cell counting

Counting of CD4 + T-cells was performed using standard procedures on Becton Dickinson FACSCalibur flow cytometer (Becton Dickinson), and defined as cells/mm^3^.

### Statistical analysis

The group comparisons were analyzed by Pearson’s chi square test or Mann–Whitney U test. Spearman correlation coefficients was calculated to determine the association between seroresponses against different polyomaviruses. Linear correlation between two variables was calculated using the Pearson correlation. All tests of significance were two-sided, and *P* < 0.05 was considered statistically significant. Statistical analysis was performed using GraphPad Prism 7.0 (GraphPad Software).

## Supplementary information

Supplementary Informations.
