# Supplementary material for: Prevalence and viral loads of polyomaviruses BKPyV, JCPyV, MCPyV, TSPyV and NJPyV and hepatitis viruses HBV, HCV and HEV in HIV-infected patients in China
Source: Sci Rep. 2020 Oct 13;10:17066. doi: 10.1038/s41598-020-74244-0 (PMC7555828; doi:10.1038/s41598-020-74244-0)
Supplement: Supplementary file 1 — Supplementary Informations. [file 41598_2020_74244_MOESM1_ESM.docx]

**Prevalence and viral loads of polyomaviruses BKPyV, JCPyV, MCPyV, TSPyV and NJPyV and hepatitis viruses HBV, HCV and HEV in HIV-infected patients in China**

Xianfeng Zhou^1, 2^, Kenji Nakashima^2^, Masahiko Ito^2^, Xiaoling Zhang^1^, Satoshi Sakai^2,3^, Changhua Feng^1^, Huabao Sun^4^, Haiying Chen^1^, Tian-Cheng Li^5^, Tetsuro Suzuki^2*^

1 The Collaboration Unit for Field Epidemiology of State Key Laboratory for Infectious Disease Prevention and Control, Jiangxi Provincial Key Laboratory of Animal-Origin and Vector-Borne Diseases, Nanchang Center for Disease Control and Prevention, Nanchang 330038, China

2 Department of Virology and Parasitology, Hamamatsu University School of Medicine, Hamamatsu 431-3192, Japan

3 Department of Molecular Biology, Hamamatsu University School of Medicine, Hamamatsu 431-3192, Japan

4 Department of Clinical Laboratory, Affiliated Infectious Diseases Hospital of Nanchang University, Nanchang 330002, China

5 Department of Virology II, National Institute of Infectious Diseases, Musashi-murayama, Tokyo 208-0011, Japan

**Supplementary Table 1.** Specific primers for detection of BKPyV, JCPyV, MCPyV and TSPyV DNAs

| Primer names | Forward | Reverse |
| --- | --- | --- |
| BKPyV-LT | 5’-CTGTCCCTAAAACCCTGCAA | 5’-GCCTTTCCTTCCATTCAACA |
| JCPyV-VP1 | 5’-TAAAGCCTCCCCCCCAACAGAAA | 5’-ACAGTGTGGCCAGAATTCCACTACC |
| MCPyV-LT | 5’-TTGTCTCGCCAGCATTGTAG | 5’-ATATAGGGGCCTCGTCAACC |
| TSPyV-LT | 5’-TTTTTGGCTTTGGGGGGGTGGCAG | 5’-TGGGGAGAATGCTTTTGTTACAAATGCTACTGTGC |

**Supplementary Figure 1.**


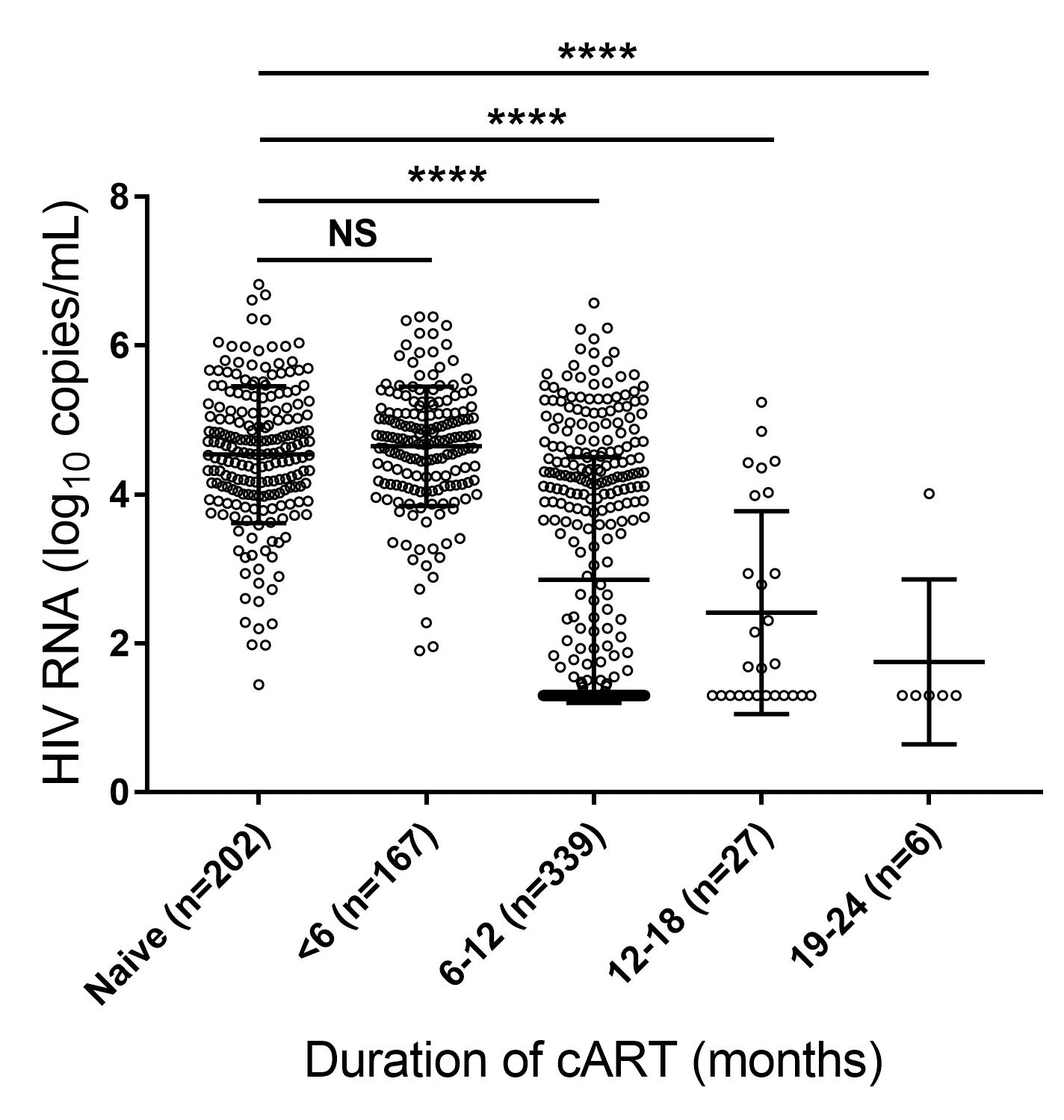


A

B


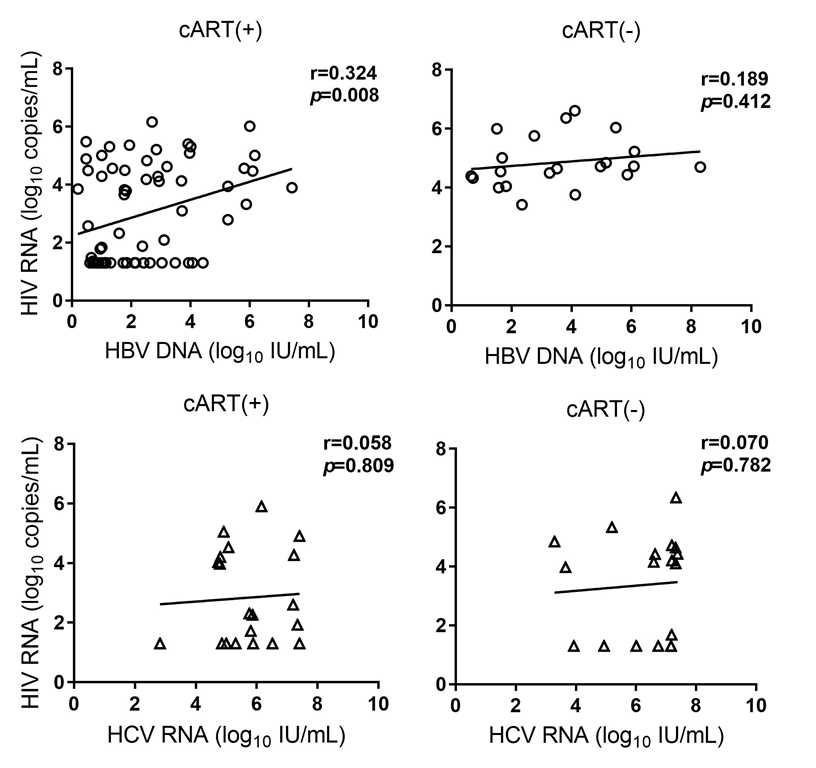


Effect of cART on the viral loads of HIV, HBV and HCV. **A**: HIV RNA levels (mean ± SD) among patients treated with cART for different durations. Mann-Whitney U test was used for group comparison. **B**: Correlation of HBV DNA and HIV RNA levels (circle); HCV and HIV RNA levels (triangle) in sera of cART(+) and cART(-) HIV/AIDS patients. Pearson correlation test was used for correlation analyses. (NS: not significant, ****: P<0.0001)

**Supplementary Figure 2.**


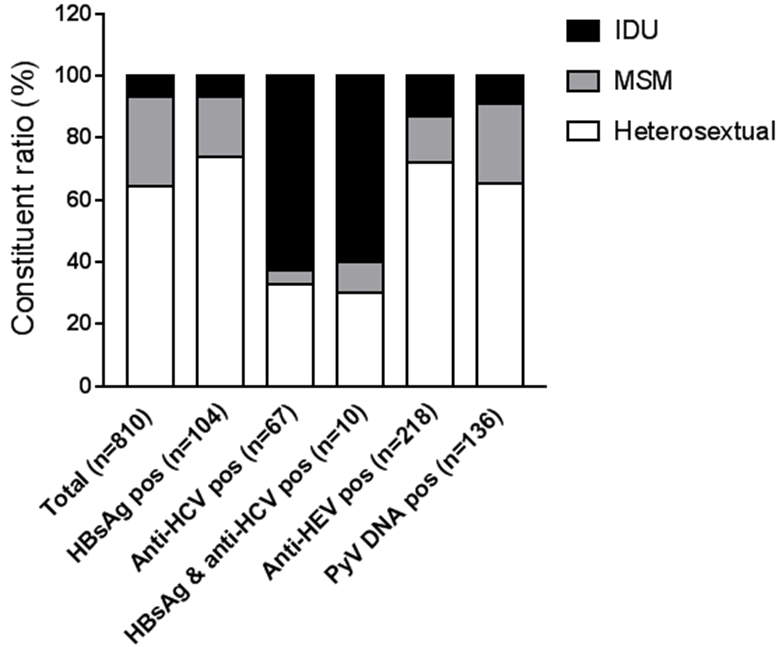


Constituent ratio of HIV infection routes in patients with different viral status. MSM: men who have sex with men; IDU: Injection drug users; Pos: positive

**Supplementary Table 2.** Seroresponses correlation between tested polyomaviruses. Gray square: HIV-positive population; White square: HIV-negative population; Spearman rank correlation test was used to evaluate the seroresponse correlation between polyomaviruses tested.

|  | **BKPyV** | **JCPyV** | **MCPyV** | **TSPyV** | **NJPyV** |
| --- | --- | --- | --- | --- | --- |
| **BKPyV** | 1 | 0.138 | 0.205 | 0.229 | 0.028 |
| **JCPyV** | 0.097 | 1 | 0.240 | 0.118 | -0.023 |
| **MCPyV** | 0.132 | 0.205 | 1 | 0.155 | -0.005 |
| **TSPyV** | 0.259 | 0.180 | 0.111 | 1 | 0.017 |
| **NJPyV** | -0.032 | -0.029 | -0.060 | -0.012 | 1 |

**Supplementary Figure 3.**


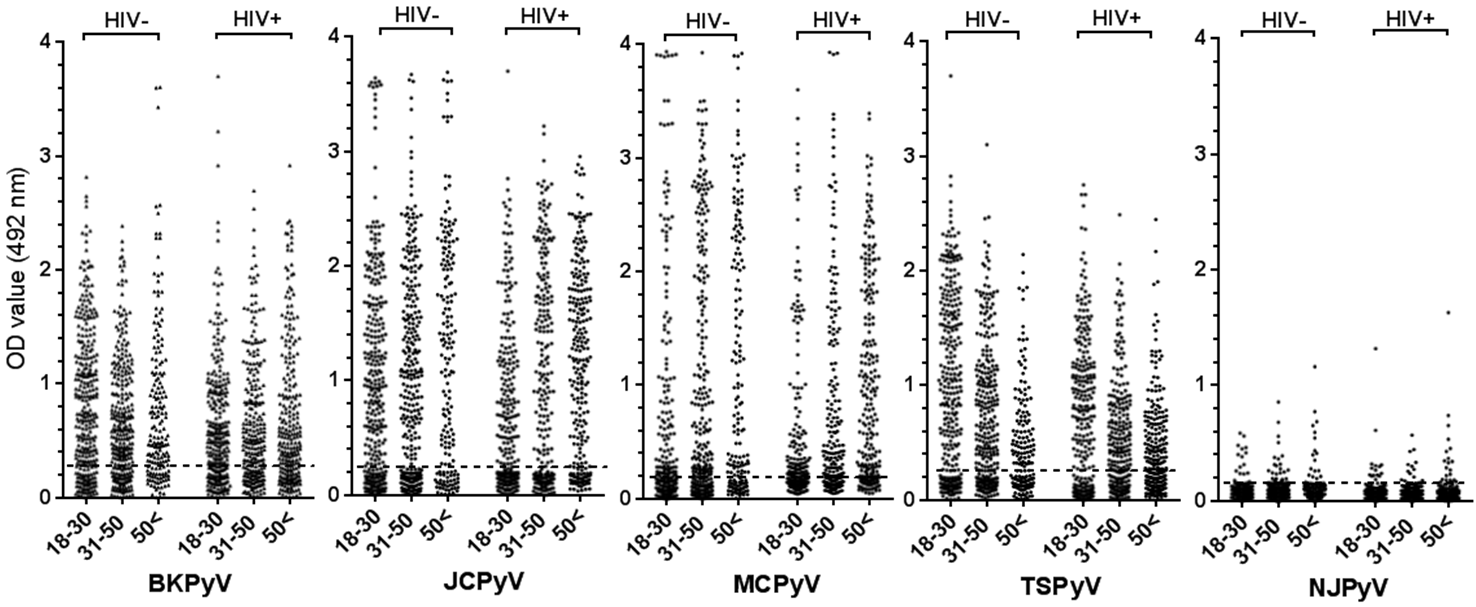


Optical density (OD) values of anti-BKPyV, -JCPyV, -MCPyV, TSPyV and NJPyV IgG antibodies in different age groups of the entire study cohort. Dotted lines represent cut-off values.

**Supplementary Figure 4.**


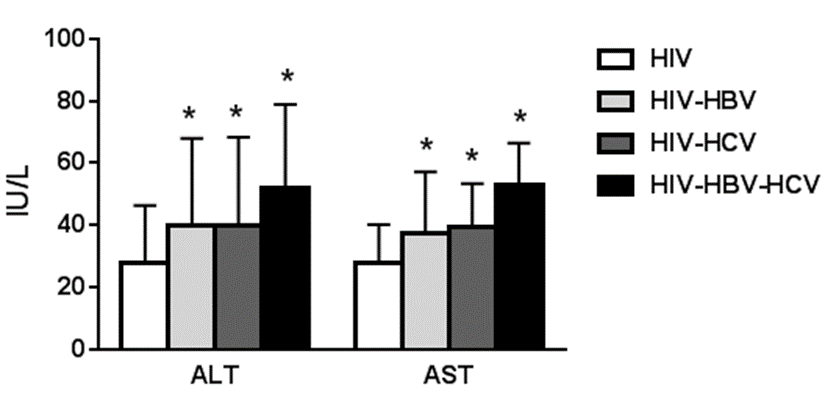


Comparison of liver function in HIV-monoinfected patients and HIV/AIDS patients co-infected with HBV or/and HCV. Serum biomarkers for liver function such as alanine transaminase (ALT) and aspartate transaminase (AST) and platelet counts were determined using OLYMPUS automatic biochemical analyzer (OLYMPUS).

Mann-Whitney U test was used for group comparison, Data represent mean with SD. (**P*<0.05, in comparison with HIV monoinfection)

**Supplementary Table 3.** Liver fibrosis evaluation of study participants; HIV monoinfection, HIV-HBV- or HIV-HCV co-infection. Two non-invasive markers, the fibrosis-4 score (FIB4) and AST-to-platelet ratio index (APRI), were used to evaluate the hepatic fibrosis of study participants. FIB4 and APRI were respectively calculated according to formulas as follows: (actual AST value divided by its upper normal limit considered as 40 IU/L)/platelet counts (10^9^/L) X 100 and [age (years) X AST (IU/L)]/[platelet counts (10^9^/L) X (ALT IU/L)^1/2^].

|  | HIV  n=426 | HIV-HBV  n=68 | P value* | HIV-HCV  n=31 | P value* | P value^#^ |
| --- | --- | --- | --- | --- | --- | --- |
| FIB-4 |  |  | 0.006 |  | <0.001 | 0.21 |
| Class1: <1.45 | 269(63.1) | 31(45.6) |  | 10(32.3) |  |  |
| Class2: 1.45-3.25 | 115(27.0) | 21(30.9) |  | 16(51.6) |  |  |
| Class3: >3.25 | 42(9.9) | 16(23.5) |  | 5(16.1) |  |  |
| APRI |  |  | <0.001 |  | <0.001 | 0.19 |
| Class1: <0.5 | 314(73.7) | 36(52.9) |  | 12(38.7) |  |  |
| Class2: 0.5-1.5 | 100(23.5) | 23(33.8) |  | 18(58.1) |  |  |
| Class3: >1.5 | 12 (2.8) | 9(13.3) |  | 1(3.2) |  |  |

*P values are for comparisons with HIV mono-infection (HIV) group. ^#^P values are for comparisons between HIV-HBV and HIV-HCV co-infected groups.

**Supplementary Figure 5.**


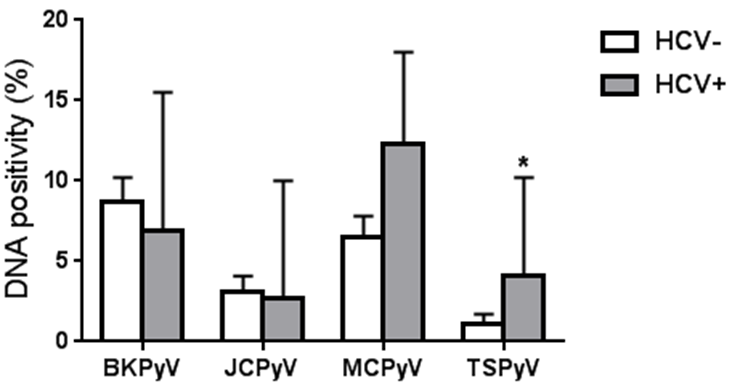


DNA positivities of BKPyV, JCPyV, MCPyV and TSPyV in anti-HCV Ab-negative (white bar) and -positive (gray bar) individuals of the entire study cohort. Pearson’s chi square test was used to compare DNA positivity between HCV- versus HCV+ groups. Error bars represent 95% confidence intervals. (**P*<0.05)

**Supplementary Table 4.** Anti-BKPyV, -JCPyV, -MCPyV, -TSPyV seroreactivities in association with different stutas of HbsAg and anti-HEV IgG in HIV-positive patients (n=810). Pearson’s chi square test was used for statistical analyses. Odds ratio (OR) with 95% CI was displayed.

| **HBsAg** | **Anti-HEV** | **Anti-BKPyV(+)** | **Anti-BKPyV(-)** | **P value** | **OR (95% CI)** |
| --- | --- | --- | --- | --- | --- |
| + | + | 28 | 9 | 0.827 | 1.090 (0.502-2.369) |
| + | - | 46 | 7 | 0.041 | 2.303 (1.016-5.224) |
| - | + | 133 | 48 | 0.881 | 0.971 (0.662-1.425) |
| - | - | 388 | 136 | / |  |
|  |  |  |  |  |  |
| **HBsAg** | **Anti-HEV** | **Anti-JCPyV(+)** | **Anti-JCPyV(-)** | **P** | **OR (95% CI)** |
| + | + | 30 | 7 | 0.071 | 2.143 (0.922-4.982) |
| + | - | 44 | 8 | 0.008 | 2.750 (1.235-5.977) |
| - | + | 132 | 50 | 0.147 | 1.320 (0.907-1.922) |
| - | - | 328 | 164 | / |  |
|  |  |  |  |  |  |
| **HBsAg** | **Anti-HEV** | **Anti-MCPyV(+)** | **Anti-MCPyV(-)** | **P** | **OR (95% CI)** |
| + | + | 30 | 7 | 0.041 | 2.346 (1.012-5.440) |
| + | - | 42 | 7 | 0.030 | 3.285 (1.448-7.450) |
| - | + | 130 | 49 | 0.048 | 1.453 (1.001-2.107) |
| - | - | 358 | 196 | / |  |
|  |  |  |  |  |  |
| **HBsAg** | **Anti-HEV** | **Anti-TSPyV(+)** | **Anti-TSPyV(-)** | **P** | **OR (95% CI)** |
| + | + | 27 | 10 | 0.355 | 1.421 (0.697-3.000) |
| + | - | 45 | 12 | 0.041 | 1.973 (1.018-3.824) |
| - | + | 115 | 66 | 0.629 | 0.917 (0.645-1.304) |
| - | - | 344 | 181 | / |  |
